# Exploring the Balance between Folding and Functional Dynamics in Proteins and RNA

**DOI:** 10.3390/ijms16046868

**Published:** 2015-03-26

**Authors:** Jovaun Jackson, Kien Nguyen, Paul Charles Whitford

**Affiliations:** Department of Physics, Northeastern University, 360 Huntington Ave, Boston, MA 02115, USA; E-Mails: jackson.jov@husky.neu.edu (J.J.); nguyen.kie@husky.neu.edu (K.N.)

**Keywords:** tRNA, molecular dynamics, biomolecular stability, ribosome

## Abstract

As our understanding of biological dynamics continues to be refined, it is becoming clear that biomolecules can undergo transitions between ordered and disordered states as they execute functional processes. From a computational perspective, studying disorder events poses a challenge, as they typically occur on long timescales, and the associated molecules are often large (*i.e*., hundreds of residues). These size and time requirements make it advantageous to use computationally inexpensive models to characterize large-scale dynamics, where more highly detailed models can provide information about individual sub-steps associated with function. To reduce computational demand, one often uses a coarse-grained representation of the molecule or a simplified description of the energetics. In order to use simpler models to identify transient disorder in RNA and proteins, it is imperative that these models can accurately capture structural fluctuations about folded configurations, as well as the overall stability of each molecule. Here, we explore a class of simplified model for which all non-hydrogen atoms are explicitly represented. We find that this model can provide a consistent description of protein folding and native-basin dynamics for several representative biomolecules. We additionally show that the native-basin fluctuations of tRNA and the ribosome are robust to variations in the model. Finally, the extended variable loop in tRNA^Ile^ is predicted to be very dynamic, which may facilitate biologically-relevant rearrangements. Together, this study provides a foundation that will aid in the application of simplified models to study disorder during function in ribonucleoprotein (RNP) assemblies.

## 1. Introduction

Over the last 10 years, it has become evident that order-disorder transitions at the molecular level are essential for biomolecular function [1]. Some notable examples include the: intrinsically disordered protein sequences facilitate DNA-binding [2]; localized order-disorder transitions (*i.e*., cracking) are associated with large-scale conformational rearrangements in proteins [3,4,5]; and the disorder of tRNA molecules can determine elongation dynamics [6]. These examples illustrate how biological dynamics result from an intricate balance of molecular stability, folding and function. 

**Figure 1:**
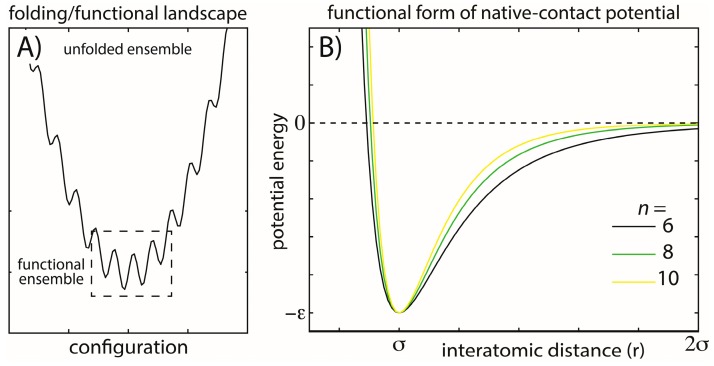
Energy landscapes and modeled energetic interactions. (**A**) Schematic view of a minimally-frustrated energy landscape for a protein folding. There is a large energy gap between the unfolded and folded ensembles, and the energetic roughness is small. Functional conformational rearrangements are associated with transitions between low-energy minima; (**B**) To explore the balance between folding and functional dynamics in biomolecules, we consider multiple functional forms of the stabilizing interactions in our model. Specifically, we change the exponent *n* associated with the attractive term in the native-contact interactions (Equation (2)). Increasing *n* has the primary effect of decreasing the length of the attractive tail and, therefore, the width of the minimum, while leaving the depth of the minimum unaltered. This specific modification was studied to determine how stability may be modulated through modifications of the entropic contribution to the free energy and whether aspects of the dynamics are robust. The explored functional form is also straightforward to implement, allowing it to be easily applied to modulate stability in other simplified and coarse-grained models.

The current study explores variations of a widely-used theoretical model for biomolecular simulations, in order to help bridge our description of biomolecular functional and folding dynamics. Specifically, we apply all-atom structure-based models (*i.e.*, all-atom “SMOG” models [7]) and determine if they can accurately capture the ratio of folding temperatures *T_f_* and physiological temperatures. In other words, can one use this approach to consistently describe protein stability and native-basin fluctuations? Structure-based models (also called Gō-like models) were originally developed for the study of protein folding, where a coarse-grained representation was employed [8]. This class of models was subsequently extended to an all-atom representation [7], which is the focus in the current study. The principle of minimal frustration states that the energetic roughness associated with protein folding is much smaller than the energy gap between the folded and unfolded ensembles (Figure 1) [9,10,11]. To approximate these minimally-frustrated landscapes, structure-based models explicitly assign each atomic interaction an energetic minimum that is consistent with the experimentally-resolved structure. In applications to proteins, models that employ these descriptions have had considerable success in elucidating many aspects of the dynamics, including the relationship between folding rates and chain length [12], dimer formation dynamics [13], the role of solvation effects [14], the influence of structure on folding mechanisms [15], mechanisms of DNA binding [16] and the stabilizing/destabilizing effects of crowding agents [17]. Using a variant of an all-atom structure-based model, long-timescale simulations have also enabled the identification of folding intermediates, predicting the modes by which proteins may aggregate and form amyloid structures [18,19].

In recent years, the use of all-atom structure-based models has been extended to RNA and large-scale biomolecular assemblies, providing insights into the physical-chemical properties of conformational transitions [20] and assembly [21] of the ribosome. When studying these larger systems, the arguments used to justify the application of these models must be considered. In the case of folding, Bryngelson and Wolynes showed that the associated landscapes are relatively smooth, or minimally frustrated [9,10,11]. This concept is central to the application of structure-based models for folding. In contrast, there is not a general principle that ensures that the landscapes associated with large-scale conformational rearrangements will be smooth. Despite the lack of an analytic theory, traditional all-atom explicit-solvent simulations of the ribosome suggest that the roughness associated with aa-tRNA accommodation is small (~1k_B_T, where k_B_ is the Boltzmann constant and T is temperature) [22]. It is important to note that since all-atom explicit-solvent simulations are typically limited to microsecond timescales, large-scale transitions are only computationally accessible in some instances [5]. Nonetheless, shorter timescale simulations (hundreds of nanoseconds) can be used to infer the magnitude of the short-scale roughness. Since the full energy landscapes of biomolecular assemblies will likely have both rough and smooth regions, it is important to consider the scope of what may be learned from applications of simpler models to conformational rearrangements. Structure-based models assign a particular configuration (or configurations) to be the dominant potential energy minimum (minima). With this “roughness-free” representation of the landscape, predicted free-energy barriers are the result of steric interactions, changes in configurational entropy and mutually-exclusive (competing) stabilizing interactions. Accordingly, these simple models may be used to identify signatures of disorder and molecular flexibility during functional conformational rearrangements.

In the current study, we consider simple variations of our class of structure-based models and ask if we can improve the model’s description of the balance between functional and folding dynamics. As described below, the original version of our structure-based model predicts that the folding temperature and the temperature at which functional transitions occur will differ by approximately a factor of two, which is significantly larger than estimates for real proteins. While these models generally provide a description of the folding dynamics that is consistent with experiments [23,24,25,26,27], this large separation of folding temperatures and functional temperatures may raise questions as to how accurately localized disorder events are described in low-temperature (*i.e.*, close to physiological temperatures) simulations with these models. Here, by making a modest adjustment to the model, we seek to find a balance between functional temperatures and folding temperatures that is consistent with experimental observations. Such an improved level of agreement between the simulations and experiments would bolster confidence in predictions of localized disorder with these models. 

To explore the balance between folding dynamics and functional fluctuations, we apply our model to multiple proteins, tRNAs and the ribosome. We first provide a detailed description of the folding dynamics of two small proteins: the SH3 domain of c-Src (SH3) and Chymotrypsin Inhibitor 2 (CI2). We focus on these two proteins, since they have been extensively studied previously, both theoretically and experimentally. By first showing that folding is appropriately described for these well-studied proteins, future studies may be extended to larger classes of proteins. For CI2 and SH3, we find that folding temperature and barrier height are significantly altered by changes in the model. We also find that the folding mechanisms are generally robust and consistent with experiments. We next compare the structural fluctuations about the native configurations in the simplified model to those obtained from explicit-solvent simulations. To characterize the robustness of RNA dynamics to variations in the model, we evaluated the native-basin fluctuations of multiple tRNA species and the ribosome. We show that the structural fluctuations are not dependent on the model, that there are aspects of tRNA fluctuations that are common across species and that there are distinct motions associated with the tRNA variable loop. Together, this study represents a systematic analysis of a commonly-used model, which provides a foundation for identifying and quantifying the role of disorder during large-scale transitions in ribonucleoprotein (RNP) assemblies, including those that engage tRNA molecules.

## 2. Results and Discussion

To construct a theoretical model that is capable of providing a consistent description of a molecule’s energy landscape, which spans from unfolded to functional configurations (Figure 1A), we considered multiple forms of our structure-based model. We assessed the performance of each variation of the model by evaluating the predicted dynamics of protein folding (CI2 and SH3) and structural fluctuations at physiologically-relevant temperatures. We also surveyed the dynamics of tRNA molecules and the ribosome. As described below, we demonstrate that variations in the model provide descriptions of the folding mechanisms of CI2 and SH3 that are largely consistent with results from previous theoretical and experimental studies. However, we find that the predicted folding temperature *T_f_* and free-energy barrier height are model dependent. Finally, we characterize the native-basin fluctuations of SH3, CI2, tRNA^Tyr^, tRNA^Ile^ and the 70S ribosome. Our analysis demonstrates that a single model that employs a simplified description of the energetics can accurately describe protein folding, protein dynamics and RNA dynamics, for the set of biomolecular systems considered. Thus, we provide evidence that this model may be used to consistently describe the dynamics of both the protein and RNA components of RNP machines.

### 2.1. Protein Folding Kinetics and Stability Are Model Dependent

**Figure 2:**
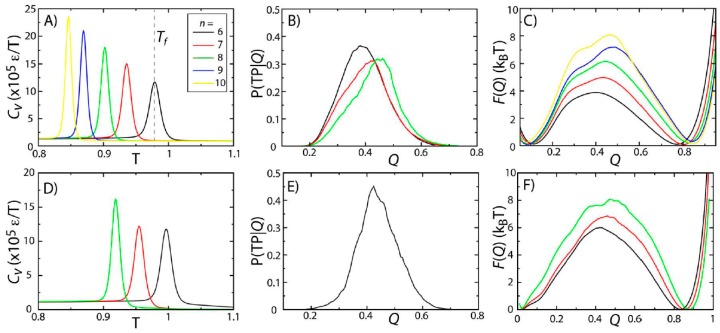
Protein folding temperatures and free-energy barriers depend on the details of the model. The folding properties of CI2 (**A**–**C**) and SH3 (**D**–**F**) were compared for different functional forms of the native contact interactions (Figure 1B). The folding temperatures, identified from the peaks in the specific heat curves (**A**,**D**), show a reduction in native-state stability as *n* is increased (*i.e.*, as the length scale of the attractive tail is decreased). For both proteins, the probability of being on a transition path as a function of the number of native contacts P(TP|*Q*) exceeds 0.3, suggesting that the transition state ensemble (TSE) is accurately captured by the coordinate *Q*. As *n* is increased, the free-energy barrier associated with folding increases. Note: Due to the magnitude of the free-energy barrier for folding of SH3, it was computationally not tractable to obtain sufficient statistics for the *n =* 9 and *n =* 10 models.

To probe the effects of model variations on protein stability and folding rates, we simulated two proteins (SH3 and CI2) using five variations of the native-contact interaction potential (Equation (2), *n =* 6, 7, 8, 9 or 10) [28]. The physical significance of changing *n* is that it modulates the length scale of the attractive tail in the atom-pair interaction. Here, we identified *T_f_* as the temperature at which the specific heat *C_v_* reaches a peak value (Figure 2A,D). For both proteins, there is a single peak in *C_v_*, consistent with folding being a pseudo-first order phase transition [15,29,30]. While the two-state character of folding is preserved across models, *T_f_* decreases with *n*. This can be interpreted as being the result of the reduced configurational entropy of the folded ensemble. That is, as *n* is increased, the native-basin is more narrowly defined, resulting in a reduction in configurational entropy and destabilization of the protein, which leads to decreased values of *T_f_*.

To study the predicted folding kinetics, we first show that our structural folding coordinate accurately captures the transition state ensemble (TSE). Here, we used the fraction of native contacts *Q* as a reaction coordinate for folding (see the Experimental Section). To evaluate the appropriateness of *Q* to describe folding kinetics, we performed long equilibrium simulations at the folding temperature and calculated the probability of being on a transition path as a function of *Q*, P(TP|*Q*). If *Q* accurately identifies the folding TSE and if the dynamics is diffusive along *Q*, then P(TP|*Q*) will reach a peak value of 0.5 [31]. This property has been used extensively by Hummer and colleagues to determine appropriate coordinates for protein folding [31,32,33,34]. Consistent with their studies, we find that *Q* performs reasonably well, where P(TP|*Q*) reaches values of ~0.3–0.4 (Figure 2B,E). This further confirms that the two-state character of the folding dynamics is preserved in all models considered here, consistent with previous theoretical and experimental measurements for these proteins [7,8,29,30]. 

While the two-state character of protein folding for CI2 and SH3 is consistently described by all variations of the model, we find that the height of the free-energy barrier is correlated with *n.* In other words, as the length scale of the attractive tail is decreased (*n* increased), the scale of the free-energy barrier is increased. This is consistent with observations from simulations with a coarse-grained model (one atom per residue), which found an increase in cooperativity of folding and larger free-energy barriers when native interactions were modeled using shorter-range interactions [35]. The same study also showed that by decreasing the length scale of the attractive tail, the distribution of folding times was more consistent with experimental ranges than when a longer-range attractive interaction was employed. While we cannot conclude on folding time distributions from our current dataset, these related findings from a coarse-grained model suggest that other aspects of protein folding may also be more accurately accounted for when larger values of *n* are used.

Since *Q* is an appropriate coordinate for describing folding of these proteins, we can infer features of the kinetics from the free-energy barriers according to $k\;=\;C\;\text{exp}(-\text{Δ}F/k_{B}T)$. ∆F is the height of the barrier along *Q*, and *C* is interpreted as a barrier-crossing attempt frequency [36,37]. Since it is not expected that *C* will vary significantly between proteins of a similar size, increased barrier heights signify a decrease in the folding rate or an increase in the mean first passage time for folding. To compare with experimental quantities, we may use a value of *C* = 1 μs^−1^ [37,38] to estimate a timescale of folding from the barrier height. A barrier of 8k_B_T (*i.e.*, the height of the barriers observed for CI2 with *n* = 10 and SH3 with *n* = 8) would correspond to a timescale ~3 ms, as compared to 60 μs for a barrier of 4k_B_T. Experimentally, these proteins have been measured to fold on millisecond timescales [29,30,39], which are more consistent with the barriers obtained with larger values of *n*. The barriers predicted by the *n* = 8 and *n* = 10 models for SH3 and CI2 are also consistent with findings from explicit-solvent simulations. Specifically, extremely-long explicit-solvent simulations predict free-energy barriers of ~4k_B_T for proteins that fold on sub-millisecond timescales [40]. Thus, the larger barriers obtained for SH3 and CI2 are consistent with these proteins folding slower than those studied using explicit-solvent models. With regards to considerations when modeling protein stability, this result suggests that a value of *T_f_* may be viewed as an adjustable parameter that can be fit by selecting the appropriate value of *n*. The resulting free-energy barrier may then be considered a consequence of the stability.

### 2.2. Robustness of Folding Mechanisms

Since structure-based models can provide descriptions of the structural characteristics of folding for CI2 and SH3 that are consistent with experimental measurements [8,23,24], we next aimed to see if the predicted mechanisms of folding are robust to changes in *n*. To describe the structural properties associated with folding, we calculated the fraction of native contacts formed with residue *i*, as a function of the global folding coordinate *Q*: *Q_i_*(*Q*). *Q_i_*(*Q*) can be interpreted as describing the order in which native structure is formed, on average. For example, if residue *i* has five contacts formed in the native configuration, then *Q_i_*(0.2) = 0.8 would indicate that, on average, four contacts are formed with residue *i* when 20% (*Q* = 0.2) of the protein’s total native contacts are formed. In comparison, one may envision a different residue *j*, where *Q_j_*(0.2) = 0.1 and *Q_j_*(0.9) = 0.8. This would indicate that, on average, the native structural content around residue *j* forms later in the folding process than the native structural content around residue *i.*

**Figure 3:**
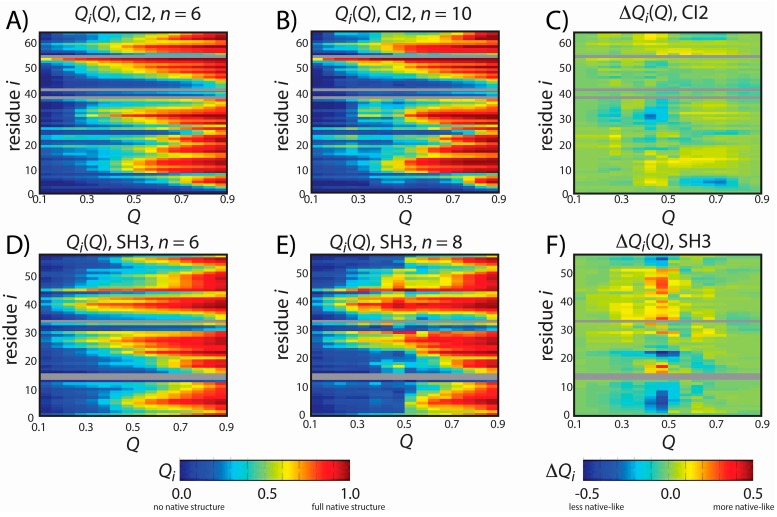
Predicted folding mechanisms of CI2 and SH3 with different models. To describe the folding mechanism, we calculated *Q*_i_(*Q*): the average fraction of native contacts formed with residue *i* as a function of the global folding coordinate *Q*. *Q*_i_(*Q*) was calculated for CI2 with (**A**) *n =* 6 and (**B**) *n =* 10 and for SH3 with (**D**) *n =* 6 and (**E**) *n =* 8. For (**A**,**B**,**D**,**E**), *Q*_i_ is colored from blue (0: no native contacts formed) to red (1: all native contacts formed). To compare the dynamics with each model (**C**,**F**), we calculated the difference in native contact formation ∆*Q*_i_(*Q*), colored blue (a decrease in native contact formation) to red (an increase in native contact formation). The order of native structure formation is robust for CI2, where ∆*Q*_i_(*Q*) is nearly zero for all values of *Q* and *i*. For SH3, ∆*Q*_i_(*Q*) adopts large values at *Q* = 0.45, indicating that there is less native structure in the tails (residues 2–7, 54–56) in the model with *n =* 8. As described in the text, this delay in the formation of the tail interactions is more consistent with experiments than the dynamics predicted by the *n* = 6 model.

The predicted structural properties of folding for CI2 are robust to changes in the energetic model. Figures 3A,B show *Q_i_*(*Q*) for CI2 with *n =* 6 and *n =* 10. To compare the folding dynamics of different models, we calculated the difference in *Q_i_*(*Q*) for the *n =* 10 and *n =* 6 models. In Figure 3C, red indicates that structure about a residue forms earlier in the *n =* 10 model than in the *n =* 6 model. Blue indicates that the native structure is formed later. Similar to an earlier study, which showed that folding mechanisms are only marginally impacted by changes in the contact strength ε [7], we find that changing the functional form of the contacts (*i.e.*, changing *n*) also has a minimal effect on the folding mechanism of CI2. 

The overall folding mechanism of SH3 is not globally altered when *n* is changed (Figure 3). However, we do find that for *Q* = 0.45–0.5 (*i.e.*, TSE region) the degree of native structure formation is noticeably perturbed. Specifically, we find that at the TSE there is less native content in the *C*-terminal and *N*-terminal tails when *n* is increased from 6 to 8. In fact, in the TSE, there is almost no native content in the tail when *n =* 8. In contrast, the *n =* 6 model predicts that approximately 30% of the native contacts are formed with the terminal residues in the TSE, similar to other theoretical results [41]. It is interesting to note that the reduced native content of the terminal residues when *n =* 8 represents an improved level of agreement with experimental measurements. That is, ϕ-value analysis is an experimental measure of the degree of native content formed about each residue in the TSE, where ϕ = 0 indicates no native content is formed and ϕ = 1 indicates all native content is formed. Previous ϕ-value measurements have shown that ϕ ~0 for the terminal residues of SH3 and ~0.8 for residues 25 and 40–50 [42,43]. These experimental values are more consistent with the results obtained with the *n =* 8 model than the results obtained with the *n =* 6 model. While the original intention of the study was not to improve the description of SH3 folding, this result does provide further support that the revised form of our model may yield a more accurate description of both folding and function.

### 2.3. Native-Basin Fluctuations Consistent with All-Atom Explicit-Solvent Models

**Figure 4:**
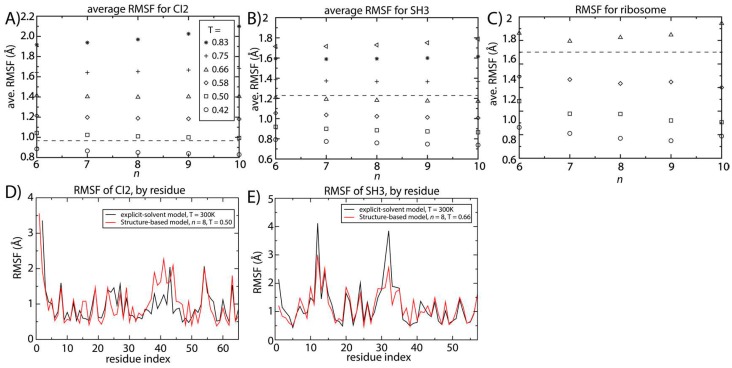
Structural fluctuations are robust to the form of the native-contact interactions. The average spatial root mean squared fluctuations (RMSF) were calculated as a function of temperature and *n* for (**A**) CI2; (**B**) SH3 and (**C**) the 70S ribosome. For all systems, the scale of the native-basin fluctuations showed a minimal dependence on the model. The average RMSF values obtained from explicit-solvent simulations are shown as dashed lines. By comparing the scale of the fluctuations in the simplified model and the explicit-solvent simulations, we identify the temperature in the simplified model that produces fluctuations consistent with those occurring at 300 K. To corroborate the character of the simulated fluctuations in the simplified model, we compared the RMSF for each residue to those obtained from two-microsecond explicit-solvent simulations of (**D**) CI2 and (**E**) SH3. There is visible agreement between the models for SH3 and CI2. For SH3, the linear correlation coefficient of the RMSF values is 0.87. For CI2, the correlation coefficient of the RMSF values is 0.65 (all residues) to 0.8 (excluding the loop region).

We next evaluated the model dependence of native-basin fluctuations in proteins and RNA. Changing the model parameter *n* has the potential to impact the fluctuations about the native configuration. That is, since the curvature of the energetic basin increases with *n* (Figure 1B), the scale of the native-basin fluctuations may decrease. Figure 4 shows the scale of the spatial root mean squared fluctuations (RMSF) as a function of temperature and *n* for SH3 and CI2. We additionally computed the RMSF for two different tRNA molecules and the ribosome. For all systems, at a given temperature, the value of the spatial RMSF only decreases slightly with increasing *n.*

To obtain benchmark estimates of the scale of molecular fluctuations at a reference temperature (*i.e*., near physiological temperature), we performed two-microsecond explicit-solvent simulations of CI2 and SH3. From these, we computed the RMSF values for both proteins. Since explicit-solvent simulations use a semi-empirical formulation of the energetics, which has been developed independently of our structure-based models, we can use these estimates of the RMSF as an external metric for assessing the scale of fluctuations in solution. That is, all-atom explicit-solvent models (CHARMM [44,45] for proteins and AMBER [46] for the ribosome) do not include explicit information about the native configuration. Rather, every atom is assigned a partial charge that approximates the local electron density, and all atom pairs interact via non-specific van der Waals interactions. Due to the increased computational requirements of explicit-solvent models, it was not feasible to perform exhaustive explicit-solvent simulations of the tRNA molecules. 

Our explicit-solvent simulations of CI2 and SH3 predict average RMSF values of 0.95 and 1.23 Å. For the ribosome, previous explicit-solvent simulations (300–1400 ns) predict an RMSF of ~1.8 Å [6]. With these values, we identified the temperature for which the structure-based model reproduces the same scale of fluctuations as observed in explicit-solvent simulations at 300 K (marked by horizontal lines in Figure 4A–C). At the temperature where the scale of the fluctuations is consistently described by both models, the distribution of fluctuations in each protein is also similar (Figure 4D,E). The correlation coefficient between the RMSF values obtained with the two approaches is 0.87 for SH3. For CI2, the correlation coefficient is smaller (0.65), where the region of poorest agreement is the extended loop (residues 35–45). Since the density of contacts is lower in the loop region, structure-based models (which are built on the contact map) are not expected to provide an optimal description of those fluctuations. When excluding the loop region, the correlation coefficient between the RMSF values exceeds 0.8, indicating agreement for regions where there is a sufficiently high contact density.

### 2.4. Identifying the Balance between Folding and Functional Temperatures

To extend the description with these models to function, we compared estimates of the folding temperature and the temperature at which native-basin fluctuations are properly described. Specifically, we determined the temperature (*T_ref_*) in the simplified model that reproduces the fluctuations observed in explicit-solvent simulations, which were performed at 300 K. Experimental measurements have implicated folding temperatures for CI2 and SH3 [29,30]. Thus, we can also compute the ratio of temperatures at which the proteins undergo native-basin dynamics *T_ref_* and the folding temperature *T_f_* in the structure-based model: *T_ref_*/*T_f_*. We compared these values to experimental estimates in order to determine how well each variant of the structure-based model can bridge the functional and folding regimes. 

**Figure 5:**
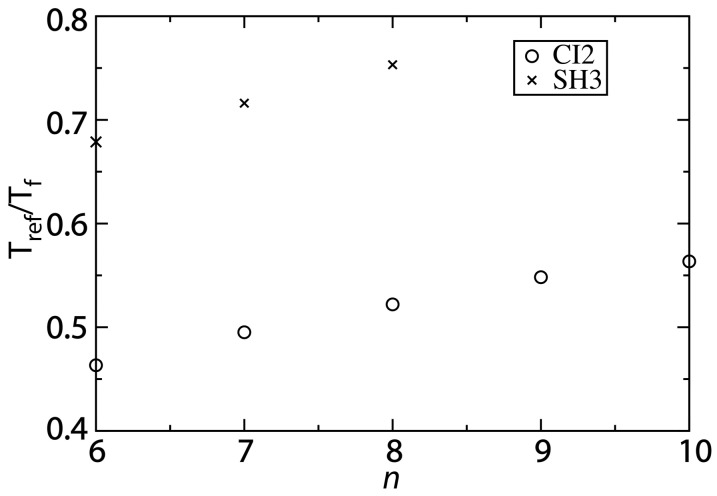
Changing the contact definition provides a description that more accurately captures the balance between folding and functional dynamics. To compare the native-basin fluctuations and folding dynamics of CI2 and SH3, we calculated the ratio *T_ref_*/*T_f_*. *T_ref_* is the simulated temperature in the structure-based model that yields a value of the average RMSF that is consistent with that obtained from explicit-solvent simulations performed at 300 K. To compare to experimental quantities, the appropriate ratio is 300/*T_f_*, where *T_f_* is the folding temperature in K. Experimentally, *T_f_* of SH3 has been measured to be ~360 K [30], corresponding to *T_ref_*/*T_f_* ~0.8. For CI2, experiments have found the folding temperature in the absence of denaturant to exceed the boiling point of water [29]. While an exact value of *T_f_* is not available, we can conclude that *T_ref_*/*T_f_* < 0.75. For both CI2 and SH3, the current study shows that increasing *n* provides a balance between folding and native-basin fluctuations in the structure-based model that is more consistent with experimental measurements.

The ratio of *T_ref_*/*T_f_* predicted by the structure-based model is more consistent with experimental observations for larger values of *n*. Experimentally, the folding temperature of SH3 has been measured to be 356 K [30]. For CI2, a precise value of the folding temperature in the absence of denaturant has been difficult to ascertain, though it is likely significantly larger than 373 K [29]. Since our description of native fluctuations is based on simulations at 300 K, the corresponding experimental values of *T_ref_*/*T_f_* can be estimated as 300/350~0.8 for SH3 and <0.75 for CI2. We find that with the structure-based model (*n =* 6), *T_ref_*/*T_f_* is approximately 0.45 for CI2 and 0.67 for SH3 (Figure 5). As already discussed, as *n* is increased, *T_f_* decreases and *T_ref_* is only marginally altered. Accordingly, increasing *n* results in increases of *T_ref_*/*T_f_* to approximately 0.6 and 0.75 for CI2 and SH3, respectively. 

While our values of *T_ref_*/*T_f_* are closer to experimental values as *n* is increased, the structure-based model may still slightly underestimate the ratio for CI2. One source of uncertainty in our analysis is that the folding temperature is known to be high, though the exact value is not known. If the folding temperature is significantly larger than 400 K, then *T_ref_*/*T_f_* will be smaller than 0.75, and it may even approach 0.6 (*i.e.*, the value obtained with the structure-based model). While there is this outstanding uncertainty, the increased values of *T_ref_*/*T_f_* predicted by the modified structure-based models (*n =* 8 or 10) represent significant improvements over earlier implementations of these models. That is, in addition to providing an accurate description of folding mechanisms and the scale of native fluctuations, when the proper variation of the model is utilized, it can also recover a balance between local fluctuations and global stability that is consistent with experimental measurements. 

It is important to note that this analysis is focused on two model proteins, CI2 and SH3. By providing in-depth analysis of two well-studied proteins, which represent two distinct folding motifs, these calculations suggest there may be a degree of generalizability to other proteins. In subsequent studies, it will be interesting to determine to what extent the relationship between folding and functional dynamics is preserved across protein families. 

### 2.5. tRNA Fluctuations Are Robust to Variations in the Model

**Figure 6:**
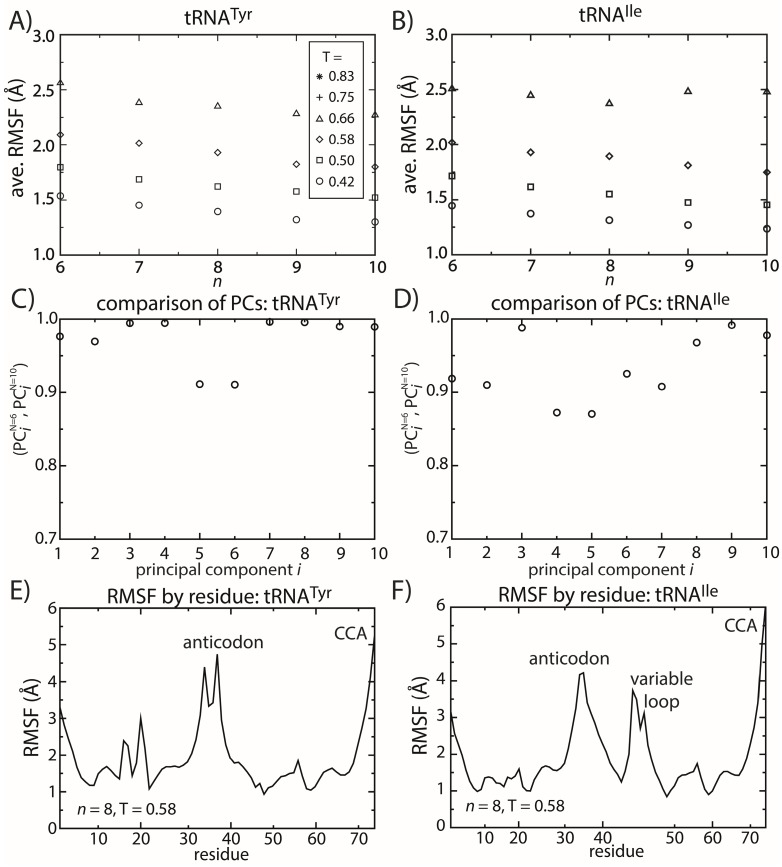
tRNA fluctuations are robust to changes in the model. The scale of the average RMSF at a specific temperature is generally consistent (within 10%) as the model (*n*) is changed. The robustness of the scale of the fluctuations is observed for both (**A**) tRNA^Tyr^ and (**B**) tRNA^Ile^. The directions of motion about the crystal structures are also robust to the model. The inner product of the *i*-th principal component (PC) when *n =* 6 and when *n =* 10 was calculated for both (**C**) tRNA^Tyr^ and (**D**) tRNA^Ile^. For the first 10 PCs, the inner product exceeds 0.9 for almost every PC of both tRNAs. The distribution of fluctuations, as measured by the RMSF of each residue, is similar between the two tRNAs (**E**,**F**). In both tRNAs, the CCA end and the anticodon region are the most mobile. The most significant difference is that the variable loop is highly dynamic in tRNA^Ile^, which is absent in tRNA^Tyr^.

Similar to the dynamics of proteins, simulations of tRNA^Tyr^ and tRNA^Ile^ show that the dominant motions are robust to the details of the model. To describe the fluctuations of tRNA molecules, we calculated the principal components (PCs) of the spatial atomic fluctuations for each tRNA species. [47] To quantify the impact of model details on the predicted dynamics, we compared the directions and scales of the motions for different values of *n*. To do so, we calculated the inner product (*i.e.*, the normalized projection) of the *i*-th principal component calculated from structure-based models with *n =* 6 and *n =* 10. The inner product can range from zero to one, where one indicates that the directions of motion are parallel and zero indicates that the motions are orthogonal. We found that for the first 10 PCs (*i.e.*, the 10 largest scale motions), the inner product exceeds 0.8 (Figure 6C,D). This confirms that the character of the dominant motions is not dependent on the precise functional form of the contact interactions. Rather, these collective motions are the result of the architecture of the tRNA molecule. 

### 2.6. Common and Distinct Properties of Different tRNA Species

tRNA molecules undergo many functional and processing steps that require the molecules to be very dynamic, including binding synthetases [48], binding elongation factors [49] and moving through the ribosome during elongation [50]. This range of roles suggests that many evolutionary pressures have contributed to the distinct shape and dynamics of each type of tRNA. To enable this versatility, each molecule has a unique sequence that can include an extended variable loop, as well as post-transcriptional modification [51,52]. While the overall fold of all tRNAs is similar, sequence extensions in the variable loop contribute to the size and charge of each molecule, which may alter the intrinsic dynamics by increasing/decreasing flexibility near the tRNA elbow region.

To elucidate possible tRNA species-specific fluctuations, we compared the simulated dynamics of tRNA^Tyr^ and tRNA^Ile^. As already discussed, the scale of the tRNA structural fluctuations is largely unaffected by the details of the model. However, comparison of the dynamics of the tRNA molecules reveals notable differences and similarities. First, we compared the RMSF of each residue for both tRNA species (Figure 6E,F). With the exception of the variable loop, the two tRNA molecules have similar RMSF profiles. Specifically, the most dynamic regions of the tRNA are the anticodon (AC), acceptor stem (AS) and CCA tail. From crystallographic structures and cryo-electron reconstructions of tRNA at various points during elongation [50,53,54,55,56,57,58,59,60], one may rationalize the biological need for a flexible AC region. Specifically, during elongation, each tRNA recognizes an mRNA sequence, interconverts between the deformed A/T configuration and a classical A/A configuration during accommodation and also transitions between numerous intermediate configurations during translocation [61,62]. At each step, the AC region can adopt distinctly bent configurations [50,53]. Accordingly, flexibility centered at the AC may facilitate these conformational rearrangements. Similarly, the flexibility of the AS and CCA regions is necessary for tRNA to bind synthetases, elongation factor Tu and ribosomal binding sites. 

**Figure 7:**
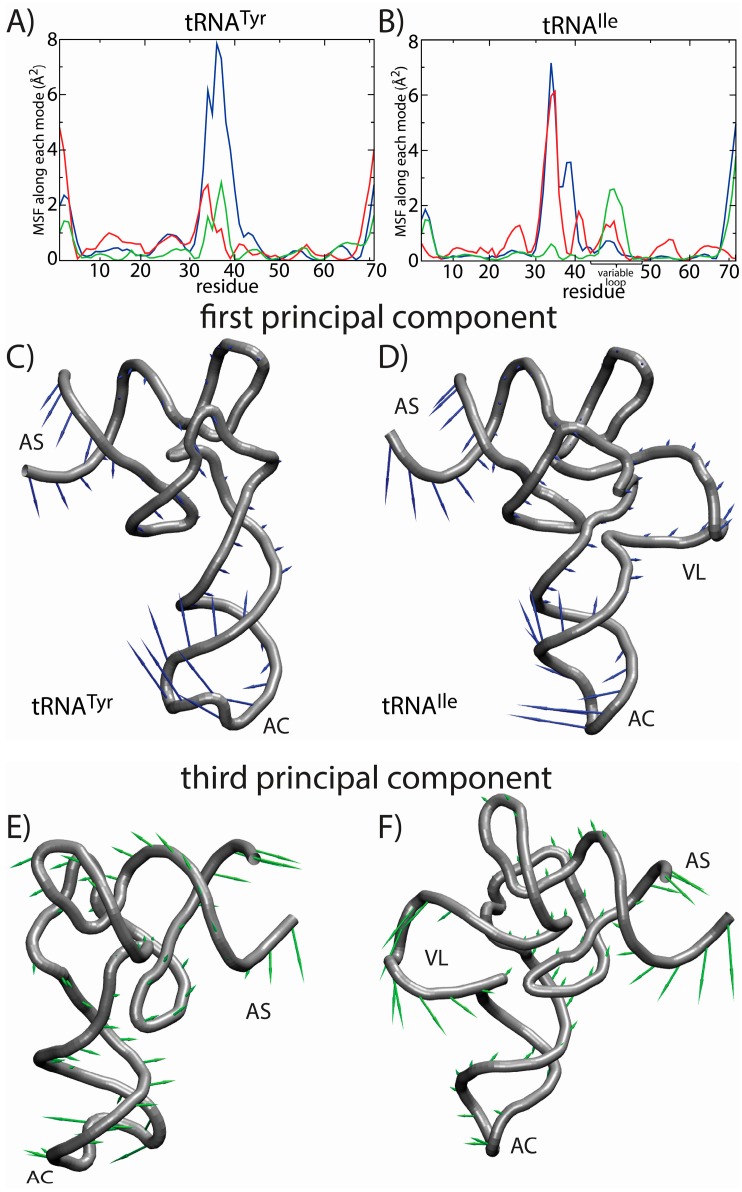
Principal component (PC) analysis reveals the correlated dynamics of the tRNA variable loop and acceptor stem (AS). To elucidate the tRNA-specific dynamics associated with the presence of a long variable loop, we compared the native-basin fluctuations of tRNA^Tyr^ (left) and tRNA^Ile^ (right). The mean squared fluctuations (MSF) of each residue along PC *i* is shown for the first three PCs (1, blue; 2, red; 3, green) for (**A**) tRNA^Tyr^ and (**B**) tRNA^Ile^. In tRNA^Tyr^, all three PCs describe the motion of the anticodon (AC) region. For tRNA^Ile^, the first PC describes the motion of the AC region, whereas the second and third PCs describe a combination of AC, AS and variable loop (VL) motions. Of particular interest is the fact that the third mode shows clear correlated movement of the AS and VL, suggesting that the VL may be actively engaged during elongation-related conformational rearrangements. The relative movement of each residue in each mode is depicted by arrows for the first PC (**C**,**D**) and the third PC (**E**,**F**). The first PC is visibly similar for the two tRNA molecules. For the third PC, the directions of the motion of the AS region are similar. However, in tRNA^Tyr^, the AS motion is correlated with the movement of the AC, and in tRNA^Ile^, the AS motion is correlated with the movement of the VL. Structural figures were prepared using VMD [65].

The most significant difference in the RMSF profiles of the two tRNA molecules is centered at the variable loop (VL) residues of tRNA^Ile^. This region is structurally significant, since it represents a nine residue-long insertion at position 47, relative to the sequence of tRNA^Tyr^. The fluctuations of the variable loop are comparable in scale to the fluctuations of the AC, AS and CCA tail. Since fluctuations in the AC, AS and CCA end have clear functional relevance, it is possible that the dynamic nature of the VL may also fulfill previously unidentified biological roles. To compare the characteristics of the dominant motions of the tRNA molecules, we visualized the first PCs and plotted the amplitude of motion of each residue along each PC (Figure 7). Here, we focus on the first several PCs, since they describe the largest scale motions. Inspection of the first PC illustrates that the motions are qualitatively similar, where both tRNA species undergo bending motions (Figure 7C,D). Specifically, this type of rearrangement is characterized by large correlated displacements of the AS/CCA and AC regions. Previous computational studies predicted similar motions and showed that these intrinsic tRNA fluctuations are often along directions consistent with conformational rearrangements during elongation [63,64]. Interestingly, large-scale motion of the VL is implicated in the second and third PCs. Further, the motion of the VL along the third PC is correlated with rearrangements in the AS, but not the AC. Since the intrinsic fluctuations of tRNA molecules facilitate functionally-relevant conformational changes inside of the ribosome [64], the observed correlation between VL and AS movements suggests that the VL may also be an active contributor to elongation dynamics. While simulations of tRNA in complex with the ribosome would be required to identify the precise role during elongation, the correlation between VL and AS suggests that there will be pronounced differences in the elongation dynamics of different tRNA species.

## 3. Experimental Section

### 3.1. Structure-Based Models

To help establish a theoretical model that can bridge folding and functional dynamics, we explored a class of simplified models. Using these models, we assessed which aspects of the predicted dynamics are robust to minor changes in the model. The only modification that we considered was the functional form of the stabilizing native interactions (Figure 1B). In this model, any two atoms that are in contact in the native configuration are treated as stabilizing. It is important to note that the energetics in this model represent effective energies. That is, the atomic interactions formed in the native configuration are effectively stabilized, since the potential of mean force necessarily has a minimum at that interatomic distance. While the position of the minimum is well defined, there is some degree of flexibility in the functional form of the interaction [66]. In our original implementation of this model [7], we employed a potential energy function of the form:

(1) $$V_{contact}\;=\;\frac{A}{r^{12}}-\frac{B}{r^{6}}$$

where *A* and *B* are assigned values, such that the minimum potential energy is at a distance σ (*i.e.*,the native distance of the atom pair), and the depth is −ε (Figure 1B). Native contacts were determined using the Shadow algorithm [67]. The Shadow algorithm defines an atom pair as a native contact if they are separated by less than 6 Å in the native configuration, they do not have a third atom in between them (shadowing a radius of 1 Å) and the atoms are separated in sequence by at least 3 (in proteins) or 1 (in RNA) residues. Bond lengths and angles are described by harmonic interactions. This ensures that the chemical structure is maintained. Dihedral angles are assigned four-body cosine interaction terms. A full description of the model can be found elsewhere [20]. Atomic models were obtained from the PDB database: CI2 (1YPA) [68], SH3 (1FMK) [69], tRNA^Ile^(1H3E) [70], tRNA^Tyr^(1FFY) [71]. 

All simulations that employed structure-based models were performed using the GROMACS software package [72,73], and input files were generated using the structure-based model web tool [74]. Since structure-based models are not part of the GROMACS distribution, the required .top (force field) and .gro (initial coordinate) files were generated by the smog-server web tool [75]. These input files are formatted for use with GROMACS v4, allowing one to integrate the equations of motion and utilize all simulation protocols and sampling techniques supported by GROMACS.

Generally speaking, many organisms live at temperatures ranging from 300 and 400 K, and folding temperatures of biomolecules are often between 350 and 450 K. Thus, one can estimate that an organism will function at a temperature that is roughly 70%–80% of the folding temperature of its constituent molecules. As discussed in the Results section, the original structure-based model yields a larger separation in relative temperatures. 

Since the original model underestimates the ratio of functional temperatures [76] and folding temperatures, we were motivated to explore variations of the model that could reduce the folding temperature. Specifically, we considered SMOG models, where the native contacts were described by a potential energy of the form:

(2) $$V_{contact}\;=\;\frac{A_{n}}{r^{12}}-\frac{B_{n}}{r^{n}}$$

For a given simulation, a specific value of *n* was employed (*n =* 6, 7, 8, 9, 10). Consistent with the earlier implementation of the model, *A_n_* and *B_n_* are set such that the position and depth of each interaction (σ, ε) are independent of the choice of *n*, which ensures that the potential energy of the folded configuration is unaltered by this modification. However, the width of the energetic minimum decreases with increasing *n* (Figure 1B), which is expected to alter the configurational entropy of the folded ensemble. Specifically, an increase in *n* should lead to a decrease in configurational entropy, which destabilizes the protein and reduces the folding temperature. In addition, increasing *n* may also reduce the scale of native-basin fluctuations at a given temperature, though this effect is found to be minimal. Together, these changes should bring the two temperature scales more close together, which would represent an improved level of agreement with experiments.

### 3.2. Structure-Based Model Simulation and Analysis Details

#### 3.2.1. Folding Simulations

To sample the full folding/unfolding space, we performed numerous unbiased constant temperature simulations of each protein. Temperatures were chosen such that at low temperatures, the proteins remain folded and at high temperatures, the proteins remain unfolded. Around the folding temperature, the proteins were observed to spontaneously fold and unfold repeatedly. A time step of 0.002 (reduced units) was used. Each simulation was performed for 1 × 10^9^–4 × 10^9^ time steps. A constant temperature was maintained through the use of Langevin dynamics. To characterize the folding mechanism, a single long folding simulation was performed for each parameter set, at the corresponding folding temperature.

#### 3.2.2. Contact Analysis and Transition Path Analysis

To quantify the folding events, we probed the fraction of native contacts formed, as a function of time *Q*(t). A contact between two residues was considered formed if any of the atomic interactions were within 1.5-times the native distance, consistent with earlier definitions of *Q*(t) [7]. To describe the folding mechanism, we used *Q*(t) from a long simulation at the folding temperature of the protein. We also used *Q*(t) to calculate the probability of being on a transition path, as a function of *Q*: P(TP|*Q*). In order to calculate P(TP|Q), we first identified all transition events. A transition event was defined as occurring when *Q*(t) adopted a value of *Q*_unfolded_ and then reached *Q*_folded_ before returning to *Q*_unfolded_. *Q*_unfolded_ and *Q*_folded_ were defined as the values of *Q* for which the free energy has minima corresponding to the unfolded and folded ensembles. Thermodynamic quantities (free energies and the specific heat) were evaluated using the Weighted Histogram Analysis Method algorithm [77,78]. With the transition events identified, P(TP|Q) was defined as the number of sampled configurations at a given value of *Q* that were part of a transition event, divided by the total number of times the system adopted the value *Q*. The number of folding/unfolding events observed for CI2 were 168 (*n =* 6), 69 (*n =* 7), 19 (*n =* 8), 16 (*n =* 9) and 5 (*n =* 10). For SH3, the number of folding/unfolding events observed were 28 (*n =* 6), 10 (*n =* 7) and 7 (*n =* 8). Due to the slower kinetics with increasing *n*, fewer events were observed for larger values of *n*, limiting the evaluation of P(TP|Q) to smaller values of *n*.

### 3.3. All-Atom Explicit-Solvent Simulations

To identify the scale of structural fluctuations at 300 K, we performed all-atom explicit-solvent molecular dynamics (MD) simulations of CI2 and the SH3 domain of c-Src Kinase. MD simulations were performed with the GROMACS-v4.6.1 software package [72,73]. The CHARMM27 force field [44,45] and TIP3P water model [79] were used. Each protein was solvated in a triclinic box with a 10 Å buffer on all sides. Counter ions (either Cl^−^ or Na^+^) were introduced to neutralize the charge of the system. Energy minimization was performed using the steepest descent and conjugate gradient algorithms. Equilibration simulations were performed using constant number-volume-temperature (NVT, 2 ns) and constant number-pressure-temperature (NPT, 10 ns) ensembles. During both equilibration phases, harmonic position restraints were imposed on all non-hydrogen atoms. To ensure constant temperature, the Nose–Hoover thermostat [80,81] was employed with a relaxation time of 0.5 ps. The Parrinello–Rahman barostat [82] was used to ensure constant pressure (1 bar), with a relaxation time of 2.5 ps and compressibility of 4.5 × 10^−5^/bar. Using the Verlet integration scheme [83] and periodic boundary conditions, we performed the NPT production runs for 2 µs, with a time step of 2 fs. Bond constraints were imposed though use of the Linear Constraint Solver (LINCS) algorithm [84]. The cutoff distances for both van der Waals and Coulomb interactions were set to 10 Å. Long-range electrostatic interactions were evaluated using the particle mesh Ewald (PME) method [85], with a Fourier spacing of 1.2 Å, an interpolation order of 4 and a tolerance of 1 × 10^−5^. The first 100 ns of each simulation were considered equilibration and were not considered in the data analysis.

## 4. Concluding Remarks

As we continue to develop our understanding of biological assemblies, including RNP machines, an exciting theme is that biomolecular disorder is central to functional dynamics. This indicates that there is a precise balance between biomolecular stability and conformational fluctuations. In the current study, we have presented advances towards establishing a class of theoretical models that can properly describe the balance between biomolecular folding dynamics, stability and folded-state dynamics. We additionally applied these models to study native-state fluctuations in tRNA molecules, which revealed that the fluctuations are largely robust to changes in the energetic model. This suggests that the secondary and tertiary structure is a major determinant of biomolecular fluctuations. We further applied this model to multiple tRNA species, which demonstrated the active role that the extended variable loop may have during functional rearrangements of tRNA molecules. Together, the robustness of tRNA dynamics and the accurate description of the stability and folding dynamics of proteins demonstrate that this model may now be used to provide more reliable predictions of disorder during conformational rearrangements in large-scale biomolecular assemblies. 
